# Anti-Immune Strategies of Pathogenic Fungi

**DOI:** 10.3389/fcimb.2016.00142

**Published:** 2016-11-15

**Authors:** Caroline M. Marcos, Haroldo C. de Oliveira, Wanessa de Cássia M. Antunes de Melo, Julhiany de Fátima da Silva, Patrícia A. Assato, Liliana Scorzoni, Suélen A. Rossi, Ana C. A. de Paula e Silva, Maria J. S. Mendes-Giannini, Ana M. Fusco-Almeida

**Affiliations:** Laboratório de Micologia Clínica, Departamento de Análises Clínicas, Faculdade de Ciências Farmacêuticas, Univ Estadual PaulistaSão Paulo, Brasil

**Keywords:** pathogenic fungi, immune response, host-pathogen interaction, fungal immune evasion mechanisms, fungal infection

## Abstract

Pathogenic fungi have developed many strategies to evade the host immune system. Multiple escape mechanisms appear to function together to inhibit attack by the various stages of both the adaptive and the innate immune response. Thus, after entering the host, such pathogens fight to overcome the immune system to allow their survival, colonization and spread to different sites of infection. Consequently, the establishment of a successful infectious process is closely related to the ability of the pathogen to modulate attack by the immune system. Most strategies employed to subvert or exploit the immune system are shared among different species of fungi. In this review, we summarize the main strategies employed for immune evasion by some of the major pathogenic fungi.

## Introduction

The increasing occurrence of fungal infectious diseases represents a major challenge for human health worldwide. It is estimated that the total number of fungal species exceeds 1.5 million (Hawksworth, 2001), and among these species, more than 600 are reported to be capable of infecting humans and animals, causing simple to fatal infections (Brown et al., 2012b). These infections lead to a wide range of diseases that include allergies, superficial infections, and invasive mycoses (Denning and Bromley, 2015), which are often associated with high rates of morbidity and mortality (Post et al., 2007). The global burden of fungal diseases has increased in parallel to the increased number of patients with human immunodeficiency virus, cancer, receiving immunomodulatory therapy, and receiving transplants, as well as premature neonates and the elderly (Vallabhaneni et al., 2015). Furthermore, the Global Action Fund for Fungal Infections (GAFFI) estimated that ~1.5–2.0 million people die of a fungal infection each year, surpassing those killed by either malaria or tuberculosis (Denning and Bromley, 2015).

The host innate immune defense of immunocompetent patients is capable, in general, of systematically eradicating opportunistic fungal pathogens. However, in immunocompromised hosts, the fungus can more easily evade detection by host defense components and eventually establish ensuing disease (Hage et al., 2002; Chai et al., 2009).

The outcome of a fungal infection often depends on the status of the host immune system, which is the first line of defense against foreign pathogens. However, patients suffering from a weakened immune system are more susceptible to the development of a serious fungal infection (Romani, 2004; Becker et al., 2015), which can progress to a very serious condition with the known ability of pathogenic fungi to rapidly adapt and become resistant to antifungal agents (Vermeulen et al., 2013). These features provide researchers with an important challenge to better understand how infection occurs and how we can prevent the development of a mycosis that can rapidly lead to death. Host defense mechanisms against fungi are numerous, and range from protective processes that were developed early in the evolution of multicellular organisms (“innate immunity”) to sophisticated adaptive mechanisms (“adaptive immunity”), which are specifically induced during infection and disease (Romani, 2011).

The surveillance and elimination of fungal pathogens depend heavily on the sentinel behavior of phagocytic cells of the innate immune system, especially macrophages, and neutrophils (Erwig and Gow, 2016). Macrophages are essential for mediating the first steps of an effective antifungal host defense, and neutrophils are essential to eliminate the fungal invasion, as evidenced by the observation that immunosuppression with prolonged neutropenia is a major risk factor for invasive fungal infections (Becker et al., 2015). Phagocytes can develop protective mechanism against fungi, destroying them via oxidative and non-oxidative mechanisms (Machado et al., 2004; Liu et al., 2014).

Direct antifungal effectors can eliminate pathogens either through phagocytic processes targeting fungi residing intracellularly, or through the secretion of microbicidal compounds targeting non-digestible fungal elements (Becker et al., 2015). Phagocytic processes lead to the accumulation of phagocytes at the site where fungal cells interact with the host, leading to the engulfment of fungal cells, and degradation of the same within maturing phagosomes (Erwig and Gow, 2016). The innate response can provide an instructive role for cells of the adaptive immune system through the production of pro-inflammatory mediators, including chemokines, and cytokines, the induction of co-stimulatory activity by phagocytic cells, and antigen uptake and presentation (Romani, 2011).

Consequently, to avoid these host defense mechanisms, fungi have evolved sophisticated strategies to maximize their probability of surviving in the host (Romani, 2011). According to Underhill (2007), the types of immune evasion can be divided in three categories: (1) *Stealth*—by which the fungus may effectively hide themselves from detection by specific immune cells or specific immune recognition molecules; (2) *Control*—which occurs when the pathogen can specifically activate host immune inhibitory mechanisms or actively guide immune responses toward types that are not especially effective against the microorganism; and (3) *Attack*—during which the pathogen may produce molecules that specifically destroy or disable host immune defenses.

**Figure 1:**
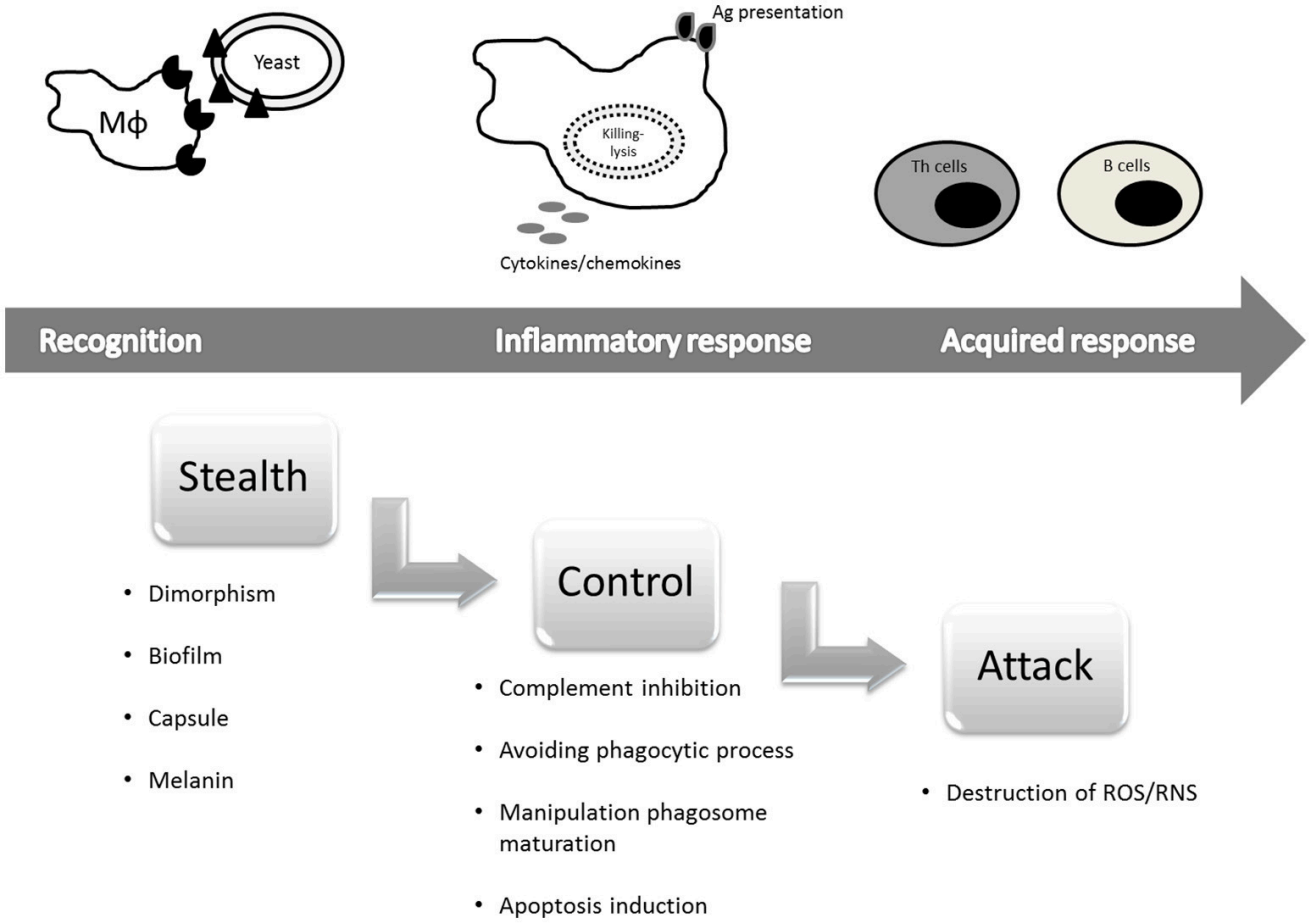
**Immune evasion mechanisms employed by pathogenic fungi divided into three categories (Underhill, 2007, with modifications)**.

Knowledge of how fungi evade the immune system can be considered analogous to a gun to fight the harmful increase in fungal infections because manipulation of the immune system could be a candidate future strategy to prevent or treat fungal infections in susceptible patients (Romani, 2011). Evasion of the host immune system is a relevant issue, and thus the objective of this review is to describe recent advances in our understanding of the mechanisms employed by the fungi to escape and efficiently infect the host by avoiding recognition by pattern recognition receptors (PRRs), modulating inflammatory signals, inhibiting complement activity, exerting anti-phagocytic mechanisms, inhibiting intracellular trafficking, and acquiring resistance to oxidative stress and antimicrobial mechanisms. This discussion is divided into three topics according to Underhill (2007) as described above (Figure 1).

## Stealth

Pathogen sensing occurs through PRRs localized in different subcellular compartments of innate immune cells, which are able to recognize conserved structures of pathogens known as pathogen-associated molecular patterns (PAMPs) that are not present in mammals (Janeway, 1989). When PAMPs are recognized, the host is “warned” of the presence of infection, initially developing a direct antifungal response through phagocytic processes followed by pro-inflammatory and antimicrobial responses through the activation of different intracellular pathways via transcription factors, kinases or adaptor molecules, leading to gene expression, and the production of cytokines and chemokines, among others (Akira and Takeda, 2004). The overall goal is to contain the infection and take up and present antigen to induce the adaptive immune system (Chai et al., 2009).

Thus, PRR recognition initiates effector and modulatory functions of phagocytic cells (Bachiega et al., 2016). PRRs are best characterized into one of four families: Toll-like (TLR), NOD-like (NLR), RIG-I-like, and C-type lectin-like receptors (CLR), each of which differ in terms of ligand recognition, signal transduction and sub-cellular localization. Most PRRs are expressed on dendritic cells (DCs) and other myeloid cells, and they are notable for initiating innate immune defenses. However, PRR signaling can also direct the development of the adaptive immune response by secreting cytokines that polarize CD4 + T cells (T-helper or Th cells; Plato et al., 2015). Different studies have demonstrated that CLRs are the major group of molecules that recognize fungi, while TRLs and NRLs play ancillary roles.

According to Underhill (2007), pathogens may shield or camouflage themselves so that they are largely ignored by the immune system. This is a simple strategy that requires the pathogen to find or produce a surface coating that is not recognized by the immune system or that is recognized but interpreted as “self” to the host.

Polysaccharides and other cell wall components are usually arranged in different layers and perform architectural and physiological functions in different locations of the cell wall. The nature of the cell wall layers of the fungi is very important for immunological detection (Erwig and Gow, 2016). Several fungal PAMPs are cell wall components, such as glucan, mannan, and chitin. Most fungi have an inner cell wall skeletal layer composed of chitin and β-(1,3)-glucan, over which other cell wall polysaccharides and glycoproteins are attached (Erwig and Gow, 2016). Some fungal species can modify chitin and β-glucan in several ways to reduce the level of host perception and thus reduce innate immune stimulation. Table 1 summarizes the host PRRs and their related fungal PAMPs.

**Table 1:** **PRR recognition of fungal components**.

**PRRs**	**Pathogen(s)**	**Fungal PAMPs**	**References**
**TOLL-LIKE RECEPTORS**			
TRL2, TRL4	*P. brasiliensis*	Unknown	Bonfim et al., 2009
TRL2, TRL4	*P. brasiliensis*	gp43	Nakaira-Takahagi et al., 2011
TRL9	*P. brasiliensis*	DNA	Menino et al., 2013
TRL2	*A. fumigatus* (conidia and hyphae form)	Unknown/α-glucan	Chai et al., 2009
TRL4	*A. fumigatus* (conidia form)	Unknown/ α-, β-glucan and galactomannan	Netea et al., 2003
TRL9	*A. fumigatus*	unmethylated CpG motifs of DNA	Ramirez-Ortiz et al., 2008
TRL4, TRL2	*C. neoformans*	glucoronoxylomannan	Shoham et al., 2001; Fonseca et al., 2010
TRL9	*C. neoformans*	CpG motif-containing DNA	Nakamura et al., 2008
TRL4	*C. albicans*	mannan (*O*-linked)	Netea et al., 2006
TRL2	*C. albicans*	phospholipomannan	Jouault et al., 2003
TRL9	*C. albicans*	CpG-oligodeoxynucleotides	Miyazato et al., 2009
TRL7	*C. albicans*	ssRNA	Biondo et al., 2012
TRL3	*A. fumigatus*	dsRNA	Carvalho et al., 2012
**C-TYPE LECTIN RECEPTOR**			
Dectin-1	*C. albicans*	β (1,3)- glucan	Gow et al., 2007
Dectin-1	*A. fumigatus*	β (1,3)- glucan	Luther et al., 2007
Dectin-1	*P. brasiliensis*	Unknown	Bonfim et al., 2009
Dectin-2	*A. fumigatus*	α-mannan	Loures et al., 2015
Dectin-2	*C. albicans*	High mannose structures	McGreal et al., 2006; Ifrim et al., 2016
Dectin-3	*C. albicans*	α-mannan	Zhu et al., 2013
DC-SIGN	*C. albicans*	High mannose structures	Cambi et al., 2003
DC-SIGN	*P. brasiliensis*	Unknown/Surface carbohydrate in extracellular vesicles/	Peres da Silva et al., 2015
DC-SIGN	*A. fumigatus*	galactomannans	Serrano-Gómez et al., 2004
Mannose receptor	*C. albicans*	mannan (N-linked)	Netea et al., 2006
Mannose receptor	*C. neoformans*	mannoproteins	Dan et al., 2008
Mannose receptor	*A. fumigatus*	mannan	Loures et al., 2015
Mannose receptor	*P. brasiliensis*	gp43	Nakaira-Takahagi et al., 2011
Mincle	*C. albicans*	Unknown	Wells et al., 2008
Galectin-3	*C. albicans*	β-1,2-mannosides	Jouault et al., 2006
Scarf1/CDC36	*C. albicans, C. neoformans*	β (1,3)- glucan	Means et al., 2009
**NRLs**			
NRLP3	*C. albicans, A. fumigatus C. neoformans P. brasiliensis*	Unknown	Gross et al., 2009; Saïd-Sadier et al., 2010; Lei et al., 2013; Tavares et al., 2013

Different complex patterns of inflammatory responses may be generated by synergism or antagonism of the stimulation of multiple receptors. Under *in vivo* conditions, an arsenal of fungal ligands is displayed in variable concentrations that result in the stimulation of different PRRs (Levitz, 2010).

β-(1,3)-glucan, the major component of the fungal cell wall, is recognized by Dectin-1 on macrophages and monocytes, inducing cytokine production and internalization of the fungus via the formation of a “phagocytosis synapse” (Brown et al., 2002). In the dimorphic fungus *Candida albicans*, the ability to grow as a filament is critical for pathogenicity (Gale et al., 1998); β-glucans are recognized by Dectin-1 only in the yeast form of this fungus, and therefore filamentous growth shields this cell wall component from Dectin-1 recognition and precludes phagocytosis and ROS production (Heinsbroek et al., 2005). Moreover, the involvement of *O*-mannan in masking β-glucan in *C. albicans* yeast cells has been demonstrated and contributes to blocking recognition by Dectin-1 (Bain et al., 2014).

Similar to Dectin-1 receptors, Dectin-2, and Dectin-3 are also transmembrane proteins from the C-type lectin family. However, while Dectin-1 recognizes β-glucans, Dectin-2 and 3 recognize α-mannan. All three Dectins are responsible for the induction of Th17 cell responses. These T helper cells (Th17) are characterized as a key response in host defense against fungi (Saijo and Iwakura, 2011).

Studies have demonstrated that Dectin-2 and Dectin-3 can form heterodimeric structures, which confer high sensitivity to the host cells with the high affinity to bind to α-mannans (Zhu et al., 2013). Recently, the role of Dectin-2 was evaluated during *C. albicans* infection, and mice deficient in Dectin-2 (Dectin-2^−/−^) are more susceptible to infection. Moreover, phagocytosis is reduced in Dectin-deficient mice, together with cytokine production. However, α-mannan detection was demonstrated by the use of *C. albicans* α-mannan and β-mannan mutants. *C. albicans* β-mannan mutants induce cytokine production differently from α-mannan mutants, and thus the authors suggest that *albicans* β-mannan can mask α-mannan and reduce recognition (Ifrim et al., 2016). A similar study using Dectin-2^−/−^ mice demonstrated the importance of Dectin-2 host defense during *C. glabrata* infection (Ifrim et al., 2014). Dectin-2 can also recognize glycoproteins containing O-linked mannobiose-rich residues present in *Malassezia* (Ishikawa et al., 2013). Thus, immune detection of intact cells initially focuses on mannan-immune response receptor interactions.

*Aspergillus fumigatus* conidia present a hydrophobic layer formed by the protein RodA and the pigment DHN-melanin, which masks β-glucans and uncharacterized TLR activators. Consequently, resting conidia do not induce cytokine release by macrophages, but during germination, the layer of RodA is degraded and molecules that are recognized by PRRs on macrophages and dendritic cells are exposed and promote cytokine production and co-stimulatory molecule expression (Aimanianda et al., 2009). Resting *Aspergillus* conidia do not present abundant β-glucans on their surface, which might account for the redundant role of Dectin-1. Inhibition of Dectin-1 on alveolar macrophages does not affect the phagocytosis of this fungus, which can be altered by the germination of conidia (Steele et al., 2005; Slesiona et al., 2012).

In dimorphic fungi, such as *Histoplasma capsulatum, Paracoccidioides* spp., and *Blastomyces dermatitidis*, the constitution of the cell wall is altered during the change in morphology; the filamentous form contains both β- and α-glucans, but conversion to the yeast form is accompanied by increased production of α-(1,3)-glucan (much less immunogenic) and has been correlated to reduced virulence that leads to the production of α-glucans, which may be a stealthy immune evasion mechanism (Borges-Walmsley et al., 2002; Brandhorst et al., 2002; Brown et al., 2003; Rappleye et al., 2007).

*H. capsulatum* secrets Eng1 protein with glucanase activity, which was shown by Garfoot et al. (2016) to be involved in the reduction of β-glucan on the yeast cell wall. Eng1-deficient yeast cells trigger increased tumor necrosis factor alpha (TNF-α) and interleukin-6 cytokine production by macrophages and dendritic cells in α-glucan-producing *H. capsulatum*. Eng1 functions in concert with α-glucan to minimize β-glucan exposure: α-glucan provides a masking function by covering the β-glucan-rich cell wall, while Eng1 removes the remaining exposed β-glucans, enhancing the ability of the fungi to escape detection by host phagocytes.

The *Cryptococcus neoformans* capsule masks recognition of the underlying cell wall mannan and β-(1,3)-glucan. Acapsular mutant strains of *C. neoformans* are readily ingested by macrophages, and both mannose and glucan receptors have been implicated in this recognition (Cross and Bancroft, 1995). Although the capsule protects the organism from recognition by phagocytic receptors (and thus is “stealthy”), it is not entirely transparent to the innate immune system. The capsule is recognized by TLRs and triggers an inflammatory response. This inflammatory response is important for restricting the growth of the pathogen during infection because TLR2-deficient mice are significantly more susceptible to *C. neoformans* infection (Yauch et al., 2004).

Another cell wall component, chitin, is covalently linked to β-glucan, and studies have shown that this component is sensed by different receptors according to the particle size and concentration, and it is involved in innate immune recognition (Shibata et al., 1997; Da Silva et al., 2010; Wagener et al., 2014). Small particles with 1–10 μm at low concentrations are able to induce production of anti-inflammatory cytokines such as IL-10 (Kogiso et al., 2011; Roy et al., 2012). Innate recognition of fungal cells by PRRs, such as Dectin-1 and TLR2, leads to the induction of pro-inflammatory cytokines such as TNF. The cytokines induce the secretion of chitinases (e.g., chitotriosidase) from neutrophils and macrophages. Chitin digestion from the cell walls of fungi by cellular activity leads to the generation of small chitin particles, which are released and taken up by the mannose receptor to induce IL-10 secretion via the TLR9 and NOD2 pathway. This mechanism may prevent inflammation-based damage during fungal infection and restore the immune balance following the clearance of infection. However, an increase in chitin particles may influence the immune system in favor of pathogenic fungal infection as a consequence of the dampened inflammatory response caused by IL-10 down-regulation of pro-inflammatory cytokine secretion (Wagener et al., 2014).

Melanins are complex amorphous polymerized phenolic compounds that are found in the inner cell walls of a wide range of dimorphic fungal pathogens. Melanin-deficient fungi have attenuated virulence because of their reduced ability to block immune recognition. Melanins prevent complement activation, neutralize antimicrobial peptides and protect cells from oxidative killing mechanisms (Nosanchuck and Casadevall, 2006). The green fungal conidial pigment dihydroxynaphthalene-melanin (DHN-melanin) of *A. fumigatus* also helps to hinder phagocytosis and conidium binding to host proteins such as fibronectin (Jahn et al., 1997; Pihet et al., 2009).

Another important feature of fungi is their ability to form a biofilm, which provides advantages in the environment and during infection. Biofilms are microbial communities that are attached to surfaces and held together by an extracellular matrix. Growth in a mass increases the resistance of the organisms to environmental stress, their resistance to antifungal activities and also effectively shields them from attack by phagocytes (Williams and Ramage, 2015). Fungi that are capable of forming biofilms are *C. albicans* (Zelante et al., 2012), *C. neoformans, H. casulatum, P. brasiliensis*, and *A. fumigatus* (Ramage et al., 2009; Pitangui et al., 2015; Sardi et al., 2015).

The nature of the immune response may be influenced by the interaction between cell wall components and host PRRs, which can be quite varied because the fungal cell wall presents interspecies and intraspecies variations. Different patterns of recognition can be observed due to the wide range of compositions of fungal cell walls and the finding that many PAMPs are shielded from their associated PRRs. Receptors that are stimulated directly influence the nature of the innate immune response and, consequently, the acquired immune response leading to different courses of fungal pathogenesis. Identification of the interaction between host receptors involved in the recognition of fungal PAMPs enables the elucidation of new mechanisms for treatment, such as the removal of sugars present on the fungal surface to promote recognition by the host or targeting more than one cell wall component for greater pathogen elimination efficiency by the host.

## Control

Stealth is not always possible, and generally an infectious organism will be recognized by the host in some manner. Successful pathogens often find ways to take advantage of host recognition systems and control them for their own means. The pathogens may exhibit on their surface or secrete molecules that specifically activate regulatory mechanisms. In this manner, the pathogen can directly inhibit the immune response or elaborate types of immune responses that are not usually effective against the organism (Underhill, 2007).

### Complement evasion

The complement system is a complex machinery that is important for innate and antibody-mediated resistance to microbial infection (Kozel, 2004). During fungal infections, many stimuli can trigger the complement pathway, and initiate an enzymatic cascade of reactions that is controlled by regulatory proteins such as foreign molecular patterns on the fungal surface, antigen-antibody complexes, and cellular debris from tissue damage promoted by inflammation associated with infection (Chai et al., 2009; Collette and Lorenz, 2011). These regulatory molecules avoid excessive inflammation and prevent tissue damage (Zipfel and Skerka, 2009).

**Figure 2:**
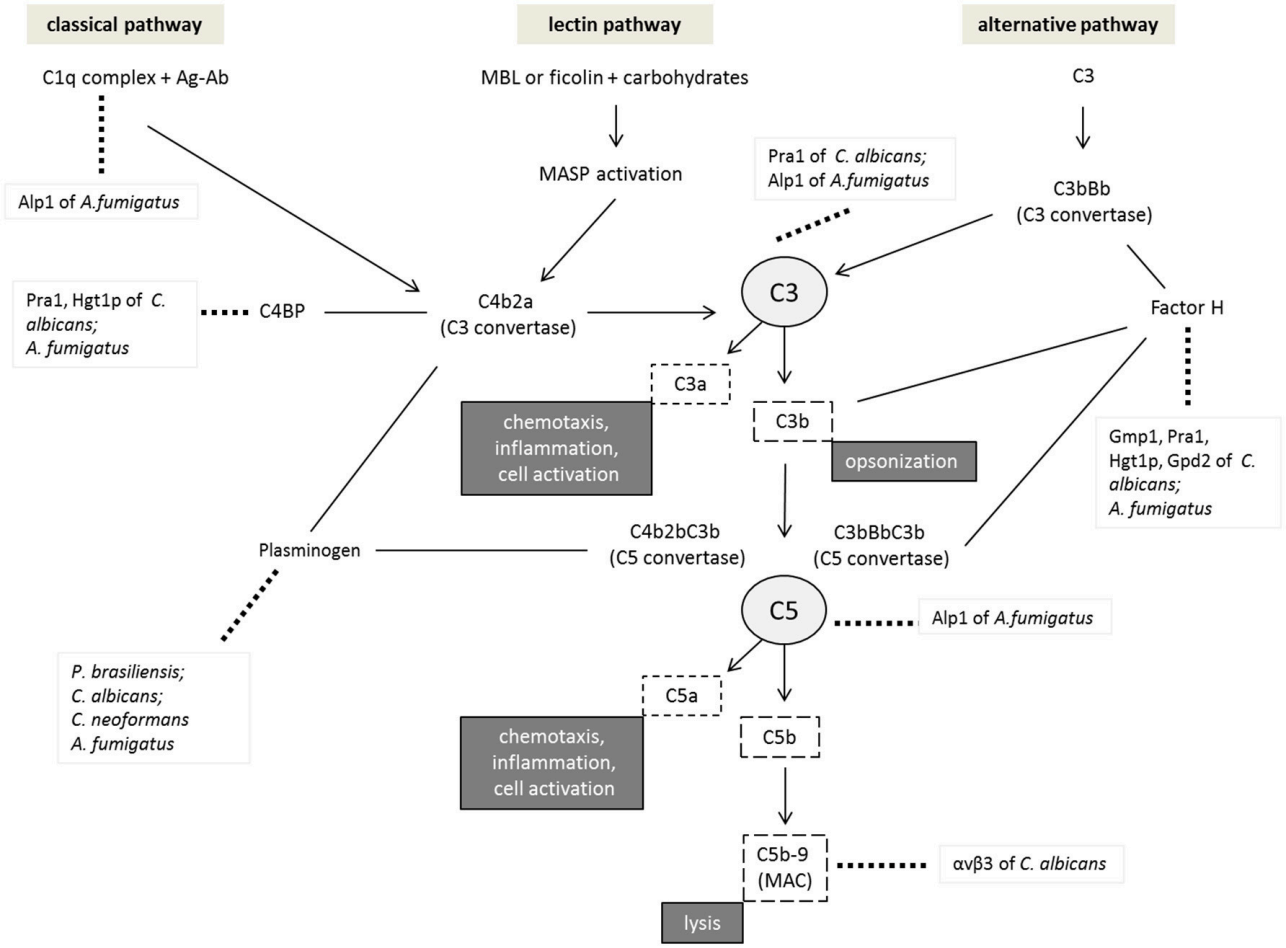
**Inhibition of the complement system by pathogenic fungi**. Activation of the different pathways is initiated by the C1q complex in the classical pathway, MBL (mannan-binding lectins), or ficolins in the lectin pathway and the C3 thioester bond in the alternative pathway. All pathways result in the assembly of the C3 convertase enzyme responsible for the cleavage of the C3 component into C3a and C3b. The binding of C3b to the available C3 convertase results in C5 convertase, which cleaves C5 into C5a and C5b. This latter binding to different components, such as C6, C7, C8, and C9 molecules, results in a membrane attack complex (MAC) that causes cell lysis. Factor H is involved in the alternative pathway as a regulatory component that prevents C3 and C5 convertase formation and inactivates C3b. Plasminogen, another protein present in plasma, is related to the complement system since it may bind to C3 and induce its cleavage into C3b and C5, inhibiting complement activation. The figure shows examples of pathogenic fungus interactions with the different components of complement system regulatory molecules or other molecules resulting from the decrease in cell lysis and opsonization favoring the survival of these pathogens.

Complement is divided into three pathways that can be activated on the pathogen surface: classical, lectin, and alternative, which differ in terms of the associated molecules or modes of initiation but converge to generate the same set of effector molecules (Janeway et al., 2001), as detailed in Figure 2.

Following activation, all complement pathways lead to the formation of C3 convertase and the C3b fragment, which results in the formation of the C5 convertase that cleaves C5 factor into C5a and C5b. This process results in a series of aggregation and polymerization steps and recruitment of the terminal components C6, C7, C8, and C9 to form the terminal complement complex (TCC). TCC is a soluble complex that is generated as a membrane attack complex (MAC) on the surface of pathogenic cells, inducing cell lysis by the insertion of C9 into the lipid layer (Speth et al., 2008; Speth and Rambach, 2012; Luo et al., 2013). However, pathogens have developed mechanisms to overcome complement attack and establish infection by, for example, binding to regulatory complement proteins, secreting proteases or avoiding opsonization.

To avoid elimination by the complement pathway, it is known that *C. albicans* and *Aspergillus* spp. secrete proteins onto their surface that bind to regulatory complement proteins. When attached to the fungal surface, these proteins inhibit the complement cascade and thus allow the evasion of complement attack (Zipfel and Skerka, 2009).

*C. albicans* has proteins that have been described as ligands for inhibitory complement proteins. Phosphoglycerate mutase (Gmp1) was the first protein described to interact with Factor H (FH) and Factor H-like protein 1 (FHL1), which are regulatory proteins of the alternative complement pathway, and plasminogen, a component of coagulation cascade (Poltermann et al., 2007).

The pH-regulated antigen 1 (Pra1) of *C. albicans* can bind to FH, FHL1, and plasminogen. In addition, Pra1 was the first protein described to bind to C4BP, which regulates the classical and lectin complement pathways and avoids C3b and C4b deposition on the fungal surface when captured by *C. albicans*, impeding complement cascade progression (Luo et al., 2009, 2011; Zipfel et al., 2013). Recently, expression of the proteins Gmp1 and Pra1 was shown to vary in clinical *C. albicans* isolates related to virulence and immune fitness (Luo et al., 2015).

*C. albicans* secretes the aspartic proteases (Saps) Sap1, Sap2, and Sap3, which degrade and inactivate the complement proteins C3b, C4b, and C5, resulting in inhibition of the damage caused by the complement system (Gropp et al., 2009). *C. albicans* Pra1 also binds to C3 and forms a complex that inhibits C3 activation, impeding complement cascade progression (Luo et al., 2010).

The high-affinity glucose transporter 1 protein (CaHgt1p) is a multifunctional protein that has been associated with evasion of the complement system by interacting with FH and C4BP (Lesiak-Markowicz et al., 2011). Glycerol-3-phosphate dehydrogenase 2 (Gpd2), another multifunctional protein secreted by *C. albicans*, plays a role in complement evasion by binding to FH and FHL1. Gpd2 also binds to plasminogen, interfering with the coagulation cascade (Luo et al., 2013).

*Aspergillus* spp. are also able to bind to the complement inhibitors FH, FHL1, and C4BP, and abrogate complement pathway progression, but how this process occurs has not been described (Behnsen et al., 2008; Vogl et al., 2008). *Aspergillus* spp. can produce enzymes that are able to degrade complement factors. Rambach et al. (2010) described a fungal protease that is able do cleave several complement components and assist fungal evasion of complement elimination during cerebral aspergillosis. Alp1 from *A. fumigatus* has also been described as a protease with broad proteolytic activity, including activity against the complement components C3, C4b, C5, and C1q, downregulating of complement cascade (Behnsen et al., 2010).

*Aspergillus* spp. use pigments on the conidial surface to mask C3 binding sites and avoid opsonization and complement attack (Tsai et al., 1997, 1998). This phenomenon has also been described for an important dimorphic pathogenic fungus, *P. brasiliensis*, which synthetizes melanin-like pigment. The melanization of yeast cells interferes with the efficiency of complement-dependent phagocytosis, avoiding interactions between components of fungal cell walls and lectin receptors on macrophages (da Silva et al., 2006). *Aspergillus* spp. also synthesize a soluble factor, complement inhibitor (CI), to inhibit complement activation and opsonization. In *A. fumigatus*, CI selectively inhibits the alternative pathway of complement and plays a role in C3-dependent phagocytosis and killing (Washburn et al., 1986, 1990; Behnsen et al., 2008).

Blastomyces adhesin 1 (BAD1), the most important virulence factor in *Blastomyces dermatitidis*, also plays an important role in complement evasion by occupying C3 sites on cell wall glucans and thus avoiding C3 deposition (Zhang et al., 2001).

*C. neoformans, C. albicans*, and *A. fumigatus* conidia can inhibit complement activity by secreting a small protein or binding several complement regulatory factors (Luberto et al., 2003; Meri et al., 2004; Behnsen et al., 2008). Additionally, the terminal MAC of the complement system is not capable of lysing the fungal cell wall (Kozel, 1996). *C. albicans* expresses an integrin called αvβ3 that acquires vitronectin to inhibit TCC formation (Spreghini et al., 1999).

Gates et al. (2004) demonstrated that the capsular matrix density and complement deposition in the *C. neoformans* capsule differ depending on whether the encapsulated yeast cells are obtained *in vitro* or *in vivo*. In the latter condition, there is a higher concentration of GXM without a significant change in the size of the capsule and with a decrease in complement deposition, which leads to reduced opsonization and poor ingestion by macrophages.

Complement activation/regulation components, such as C3, C4BP, Factors B, and H, have been shown to be responsible for 38.6% of the cell wall-bound plasma protein mass in *P. brasiliensis* (Longo et al., 2013), corroborating previous reports of immunofluorescence data showing that C3, C3a, C3d, C3g, C4, C5b-9, and Factors H and B are present on the *P. brasiliensis* yeast cell surface (Munk and Da Silva, 1992). These findings indicate that this fungus can activate the complement system, consistent with another study (Calich et al., 1979). A study comparing three isolates of *P. brasiliensis* with different degrees of virulence demonstrated differential activation of the classical and alternative pathways among the isolates. Fraction F1, an alkali-insoluble polysaccharide fraction (containing β-glucan) from low virulence isolates, was more efficient than F1 from the virulent strain for activating the complement system (Crott et al., 1997; Anjos et al., 2002).

Plasminogen is a complement regulatory protein that is present in plasma as an inactive proenzyme that can be converted (in the presence of tissue host factors) to plasmin, an active serine protease that participates in the coagulation system but that also degrades extracellular matrix (Barthel et al., 2012). The coagulation system and complement cascades are closely related (Peerschke et al., 2008); activated plasminogen is capable of cleaving complement proteins, resulting in the inhibition of complement activation.

Different fungi have plasminogen-binding proteins that bind plasminogen leading to plasmin generation and activate it to cleave complement effectors and block C3 and C5 convertase to favor C3b inactivation (Barthel et al., 2012). Examples of fungi that likely employ proteins for this evasion mechanism by binding to plasminogen are *P. brasiliensis* (Marcos et al., 2012; Chaves et al., 2015), *C. albicans* (Crowe et al., 2003; Luo et al., 2009), *C. neoformans* (Stie et al., 2009), and *A. fumigatus* (Behnsen et al., 2008), among others.

**Figure 3:**
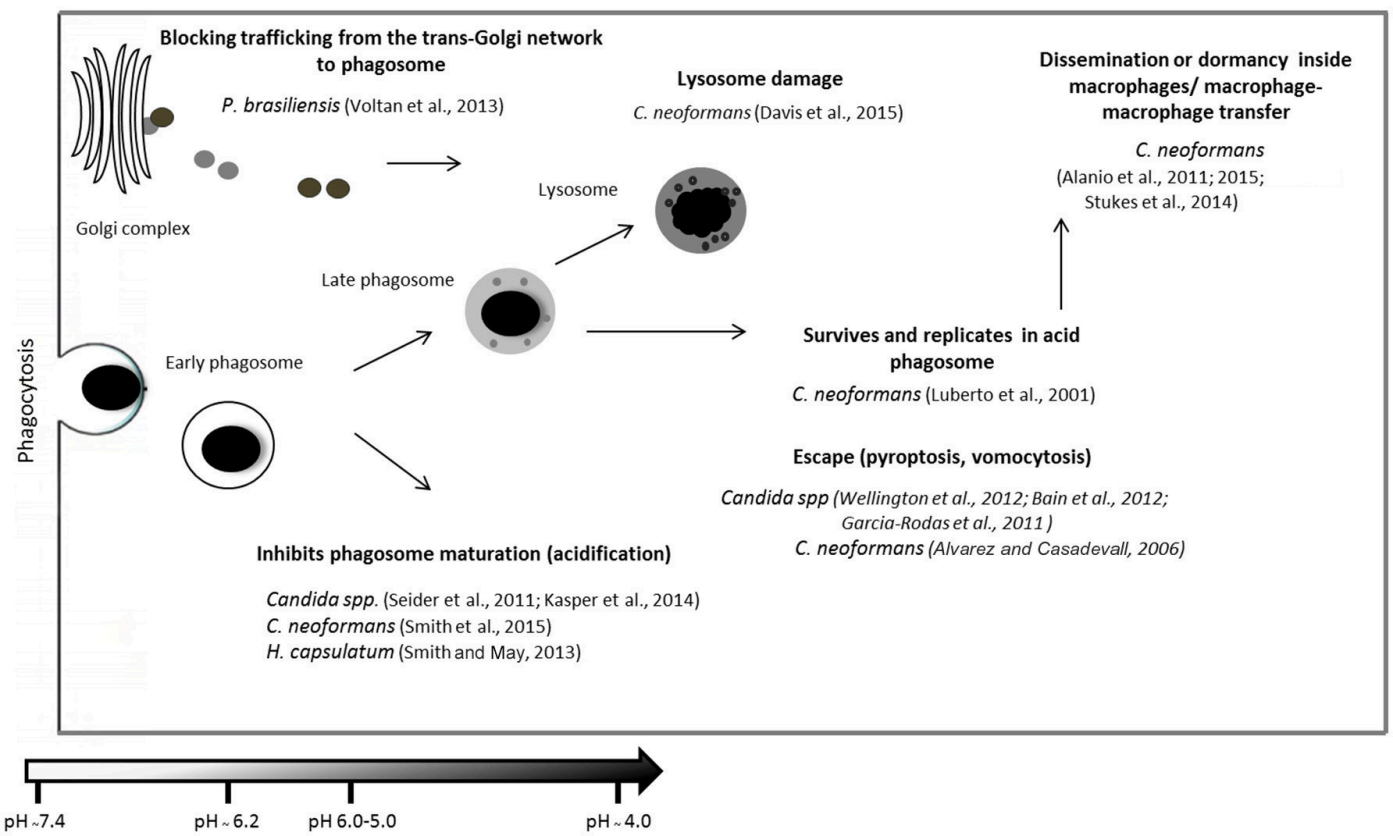
**Summary of different fungal mechanisms used to evade the immune system after phagocytosis**. Following internalization, the contents of the phagosome must be digested. Degradation is achieved by a series of phagosome maturation stages in which they receive new material from early endosomes, late endosomes, and finally lysosomes. The figure shows examples of pathogenic fungus interactions with different components of the complement system, regulatory molecules or other molecules resulting from the decrease in cell lysis and opsonization, favoring pathogen survival. Examples are shown of strategies used by pathogenic fungi to escape phagosomes and phagocytes and to alter phagosome maturation and persist within phagosomes (Luberto et al., 2001; Alvarez and Casadevall, 2006; Alanio et al., 2011, 2015; Garcia-Rodas et al., 2011; Seider et al., 2011; Bain et al., 2012; Wellington et al., 2012; Smith and May, 2013; Voltan et al., 2013; Kasper et al., 2014; Stukes et al., 2014; Davis et al., 2015; Smith et al., 2015).

Figure 3 shows a schematic of the complement activation pathways and examples of pathogenic fungi that interact with the different components of the complement system, regulatory molecules or other molecules that lead to a decrease in cell lysis and opsonization, favoring pathogen survival. The development of therapeutic approaches that interfere with fungal evasion of the complement system is highly speculative. It is very important to identify a strategy to inhibit complement activation to an appropriate extend. Inhibitors targeting the common effector phase of the complement cascade can be very efficient, but systemic inhibition of the complement system increases the risk of infections (Beinrohr et al., 2008).

Blocking fungal surface components with specific antibodies or peptides may contribute to, for example, an increase in C3 binding site exposure and therefore facilitate complement deposition and phagocyte ingestion, or block the acquisition of negative complement regulators at the fungal surface, such as FH or C4BP, potentially increasing the susceptibility of fungi to attack by the complement system. However, all these approaches are still hypothetical.

The complement system is more than simply a “guard” against pathogens. It is involved in inflammatory processes, the modulation of cellular responses, and cell-cell interactions that are crucial for early development and cellular differentiation (Mastellos and Lambris, 2002). Erroneous activation or insufficient regulation of the complement cascade may focus its destructive activity on the host cells, highlighting an obstacle in the design of complement-specific drugs. However, two complement-targeted drugs for non-fungal diseases have been introduced in the clinic: the therapeutic anti-C5 antibody eculizumab (Soliris; Alexion Pharmaceuticals) and various preparations of the physiological regulator C1 esterase inhibitor (C1-INH). In addition, several new candidate drugs targeting various components of the complement cascade are in different stages of clinical development (Ricklin and Lambris, 2013; Morgan and Harris, 2015; Reis et al., 2015; Mastellos et al., 2016).

### Escaping from phagocytic process

Once a microorganism reaches the host, the first line of defense is the phagocytic cells. Professional phagocytes (neutrophils, macrophages, and dendritic cells) of the innate immune response are responsible for controlling the infection (Qian et al., 1994). Deficient phagocytosis represents a risk factor for fungal disease. Phagocytosis is an efficient mechanism to protect the host and eliminate pathogens (Romani, 2011).

The morphology and size of the pathogen are important during phagocytosis. It is important to highlight that fungi can change morphology during different stages of infection in response to the host temperature and for dissemination (San-Blas et al., 2000; Klein and Tebbets, 2007; Boyce and Andrianopoulos, 2015). These changes increase the challenges associated with phagocytosis. The morphology will determine how the complexity of the actin filaments for successful phagocytosis. Moreover, if the microorganisms are larger than the phagocytes cells, this process may be compromised (Champion and Mitragotri, 2006). However, there are reports of macrophages undergoing division and fusion to increase cell size and win the battle against the invading pathogen (Garcia-Rodas et al., 2011; García-Rodas and Zaragoza, 2012). In contrast, dynamic studies of variously shaped and sized particles have demonstrated that shape determines the success of phagocytosis (Champion and Mitragotri, 2006; Paul et al., 2013).

Early studies have revealed that *C. albicans, Candida tropicalis, Candida krusei, Candida parapsilosis*, and *Candida guilliermondii* undergo efficient phagocytosis; however, they are also able to develop hyphae inside and outside the macrophage, multiplying intracellularly, destroying the phagocytic cell, and escaping ingestion (Stanley and Hurley, 1969).

During this transition, *C. albicans* produces hyphae within macrophages to kill the cell or outside the macrophage to avoid phagocytosis and limit the recruitment of additional macrophages (Brothers et al., 2013; Ermert et al., 2013). Moreover, yeasts, but not hyphae, are phagocytosed efficiently by macrophages. One possible explanation for this difference is that hyphal filaments contain very little glucan and do not properly activate Dectin-1 (Shoham et al., 2001; Gantner et al., 2005; Seider et al., 2010).

*C. glabrata* proliferate inside macrophages and result in cell lysis (Kaur et al., 2007; Dementhon et al., 2012). *Candida lusitaniae* escapes from macrophage activities by producing cells chains and thus avoiding recognition by the immune system (Dementhon et al., 2012). In a detailed study, Garcia-Rodas et al. (2011) showed the complexity of the *C. krusei*-macrophage interaction. They demonstrated that *C. krusei* was able to survive inside macrophages; furthermore it was capable of producing filaments and killing the macrophages.

*A. fumigatus* conidia can germinate and produce hyphae, also hindering phagocytosis (Erwig and Gow, 2016). *Paracoccidioides* spp. are thermally dimorphic fungi; at 37°C, multiple budding with irregular sizes and shapes are observed. Because of the non-uniform morphology, small cells are more susceptible to macrophage activities (Almeida et al., 2009).

The increased size of the *C. neoformans* capsule during infection confers resistance to several immune response mechanisms including phagocytosis (Maxson et al., 2007; Zaragoza et al., 2008). However, *C. neoformans* displays another feature to escape phagocytosis. Cell enlargement is an *in vivo* phenomenon, and these cells can be 5 to 10-fold larger than normal *C. neoformans* cells. These giant cells, also called titan cells, are polyploid, uninucleate, and have a thinner cell wall than normal cells. These cells are resistant to phagocytosis and oxidative stress (Cruickshank et al., 1973; Feldmesser et al., 2001; Okagaki et al., 2010). The capsule protects cells against phagocytic processes, assisting the development of disease by interfering with T cell functions (Feldmesser et al., 2000; Rodrigues and Nimrichter, 2012). The *C. neoformans* capsule comprises GXM, glucuronoxylomannogalactan (GXM Gal) and mannoproteins (MP), which trigger variations in immune responses (Doering, 2009). GXM acts as an important immune modulator by directly inhibiting the proliferation of T-cells; GXM Gal shows the ability to induce apoptosis of human T-cells, inhibiting cell-mediated immunity, and apoptosis in macrophages; MPs are immunogenic and induce the accumulation of TNF and other cytokines, such as IL-12, IL-6, IL-10, IFN-γ, and IL-8, in monocytes (Martinez and Casadevall, 2005; Li and Mody, 2010; Vecchiarelli and Monari, 2012).

Other studies have shown that GAT201, a transcription factor in *C. neoformans*, plays a role in the capsule-independent mechanism of antiphagocytosis. Deletion of this gene increases phagocytosis by macrophages (Liu et al., 2008). Luberto et al. (2003) described the role of the protein App1 in the inhibition of phagocytosis by alveolar macrophages through a complement-mediated mechanism. They used an *app1*Δ strain (with no differences in capsule size, melanin formation, or growth at 30° or 37°C) that was more easily ingested by macrophages even in immunocompetent mice that were deficient for complement C5.

Apoptosis or programmed cell death is a mechanism employed to regulate the innate immune response (Busca et al., 2009). Interestingly, some fungi can also use this mechanism as a strategy to avoid the immune system. Prevention of apoptosis, a phenomenon that can prevent phagocytosis, is helpful for microorganisms because they can protect themselves inside host cells and are protected from the cytotoxic activity of the immune system, such as secreted antimicrobial substances or immune cell attack (Ali et al., 2003; Voth et al., 2007; Volling et al., 2011).

*C. albicans* is able to induce apoptosis and use it as an evasion mechanism, and phospholipomannan has been reported to be involved in this process (Ibata-Ombetta et al., 2003). *A. fumigatus* can manipulate apoptosis, and this manipulation is morphology-dependent, in which the conidia are able to inhibit the apoptosis of different cell types (Volling et al., 2007; Féménia et al., 2009). However, during the hyphal phase, *A. fumigatus* produces gliotoxin, which is a fungal metabolite that can kill different types of cells, has anti-phagocytic activity, induces apoptosis, and consequently suppresses immune responses (Müllbacher et al., 1985; Waring et al., 1988; Sutton et al., 1994; Stanzani et al., 2005). *A. fumigatus* melanin plays an important role in fungal evasion because despite its ability to provide protection against reactive oxygen species (ROS) produced by the immune system, the melanin present in conidia is also responsible for the inhibition of apoptosis (Volling et al., 2011).

*P. brasiliensis* is also able to induce apoptosis in macrophages via the expression of caspase-2, 3, and 8; however, infection by this fungus can also induce anti-apoptotic genes (caspase-8 and Fas-L inhibitors; Silva et al., 2008). Moreover, during paracoccidioidomycosis, the fungi produces gp43, which is the main antigen detected during infection. Studies have shown that gp43 and peptides derived from this glycoprotein have the ability to avoid phagocytosis and are also considered as evasion mechanisms of this fungus (Flavia Popi et al., 2002; Konno et al., 2009).

### Surviving

Akoumianaki et al. (2016) showed that during *A. fumigatus* germination, β-glucan is exposed on the fungal surface, and this exposure leads to activation of an Atg5-dependent autophagy pathway called LC3-associated phagocytosis (LAP) that kills the fungus, but this activation requires removal of the melanin in the fungal cell wall. In this way, *Aspergillus* melanin confers resistance to killing by macrophages by inhibiting NADPH-oxidase-dependent activation of LAP by selectively excluding the p22phox subunit from the phagosome membrane; LAP blockade is a general property of fungal cell wall melanin.

*C. neoformans* has a multiple-hit intracellular survival strategy, resulting in the progressive deterioration of host cellular functions. When macrophages are infected, any cellular process can be disrupted and subsequently affect multiple cellular processes. For example, the activation of several stress pathways affects protein translation and cause mitochondrial depolarization. Mitochondrial alterations can be caused by the deregulation of cyclin D1 or, alternatively, mitochondrial alterations can potentiate endoplasmic reticulum stress. The decreased mitochondrial potential results in the deregulation of fuel and energy requirements and in poor functioning of mitochondria, which play a role in the integration of cellular decisions concerning death, survival, and immune activation, such as the activation of macrophages and their microbicidal abilities (Wagener et al., 2014). This damage results in the inability to clear the infection and facilitates the persistence of *C. neoformans* within macrophages. These inefficient immune responses rapidly lead to chronic infections (Coelho et al., 2015).

### Manipulating phagosome maturation

The phagocytosis of microorganisms and subsequent degradation of the particles internalized by phagocytic cells is a vital innate immune response to contain the dissemination of pathogens (Smith et al., 2015). Some pathogenic fungi have developed strategies to resist phagocytosis, thus increasing their pathogenicity, and survival in the host (Brown et al., 2012a). Some fungi, including *C. neoformans, C. albicans, C. glabrata, C. krusei, and H. capsulatum*, can be phagocytosed by and persist within immune cells (Eissenberg et al., 1993; Sebghati et al., 2000; Johnston and May, 2010; Seider et al., 2011).

The phagocytosis of microorganisms and subsequent degradation of the internalized particles by phagocytic cells is a vital innate immune response to contain the dissemination of the pathogen (Smith et al., 2015). Although little is known about the factors responsible for controlling phagosome maturation after yeast cell phagocytosis (Gilbert et al., 2015), pathogens utilize several approaches to prevent killing and degradation by phagocytic cells, such as inhibition of phagosome maturation or fusion, blocking phagosomal acidification, or escaping from the phagosome (Clemens et al., 2000; Deleon-Rodriguez and Casadevall, 2016).

After internalization, pathogens may be contained in the phagosome, and subsequently the maturation process is initiated (Smith and May, 2013). The fusion of the late phagosome and lysosome gives rise to the phagolysosome, in which the pH decreases to below 5.5 and hydrolytic enzymes and high levels of free radicals are together introduced to degrade the internalized pathogen or inhibit the microbial growth (Eissenberg et al., 1993; García-Rodas and Zaragoza, 2012). It is also believed that acidification is required for intracellular trafficking and antigen presentation; some pathogens have developed mechanisms to avoid the hostile low pH by modulating this pH change (Eissenberg et al., 1993).

Levitz et al. (1999) demonstrated that *C. neoformans*, in contrast to other intracellular pathogens, does not avoid fusion with macrophage lysosomal compartments but rather resides, and survives in the acidic phagolysosome. The growth of *C. neoformans* is inversely proportional to the pH; alkalization of the pH retards its growth. When the pH of the phagolysosome is artificially increased, a reduction of intracellular proliferation of the yeast was observed, indicating that *C. neoformans* has the ability to divide in an acidic pH (Luberto et al., 2001). In addition to the ability to grow under acidic pH conditions, this pathogen seems to have increased resistance to macrophage lysosomal enzymes, which require the acidic pH for their activity (Deleon-Rodriguez and Casadevall, 2016).

*H. capsulatum*, an obligate intracellular pathogen, possesses mechanisms that allow it to survive and replicate within macrophages (Inglis et al., 2013). The major mechanism is the ability to manipulate the phagosome to maintain an internal pH of 6.5, inactivating acid-dependent hydrolytic proteases and maintaining the capacity to acquire iron (a process that is usually dependent on acidification), thus favoring its replication by generating a more neutral environment (Smith and May, 2013). It is believed that this strategy involves the blockade of lysosomal fusion with the phagosome and vacuolar H+-ATPase (V-ATPase; Strasser et al., 1999), which is a large multiprotein complex that is related to the acidification process (Kissing et al., 2015).

A feature that contributes to *C. neoformans* dissemination through blood brain barrier is via Trojan horse hypothesis inside macrophages (Alanio et al., 2011), in which the pathogen takes advantage of the intracellular environment of phagocytic cells as a place to hide from direct attack by the immune system (Charlier et al., 2009; Casadevall, 2010). Inside the macrophage, the fungus can persist in the host in a state of dormancy that is resuscitated in response to the appropriate stimulus (Alanio et al., 2015). Moreover, it can escape the intracellular limitations of the macrophage in an actin-dependent manner via cell-to-cell transfer, leading to the infection of adjacent cells (Alvarez and Casadevall, 2007; Stukes et al., 2014). It is believed that this pathogen also has the ability to inhibit phagosome maturation during infection. Recently, Smith et al. (2015) used different markers of phagosome maturation to demonstrate that cryptococcal-containing phagosomes induce premature removal of Rab5 (involved in the recruitment of host effectors such as early endosome marker 1 and Rab7) and Rab11 (present on early phagosomes), thus modifying the phagosome in which it resides to alter phagosome acidification, calcium flux, and protease activity.

Analysis of the genome of *C. albicans* verified the presence of several genes that, when transcribed, allow survival in macrophages (Gilbert et al., 2015). Smith et al. (2004) found that the protein kinase Hog1p is activated by a variety of stress factors and may regulate genes in response to phagosomal conditions. Miramón et al. (2012) showed that the loss of Hog1p increases the sensitivity of *C. albicans* to killing by phagocytes.

Although, several study findings support macrophage lysis in response to the hyphal form of the fungus, Wellington et al. (2012) showed that a mutant deficient for IL-1β secretion leads to lower levels of lysis, independent of its ability to form hyphae, demonstrating that the physical formation of hyphae alone is not sufficient to trigger IL-1β secretion or macrophage lysis. This finding suggests that other processes, such as pyroptosis, a caspase-1-dependent response to intracellular pathogens, could play a role in *C. albicans*-macrophage interactions (Wellington et al., 2012). Macrophage death caused by *C. albicans* hyphae during the initial period post-phagocytosis (6–8 h) occurs by the induction of pyroptotic caspases and is dependent on caspase-1 (Uwamahoro et al., 2014) and the inflammasome subunits NLRP3 and ASC (Wellington et al., 2014). After the macrophage is destroyed via pyroptosis, it may lose its ability to release cytokines that signal for the recruitment of other immunes cells, leading to a weakened immune system.

More recently, Tucey et al. (2016) characterized the *C. albicans* endoplasmic reticulum (ER)-mitochondria tether complex ERMES (mediator of interactions between organelles, providing membrane contact sites) and showed that the ERMES *mmm1* mutant has a severely crippled ability to kill macrophages despite hyphal formation and normal phagocytosis and survival.

*C. glabrata* is able to alter phagosome maturation by blocking phagolysosome formation and phagosome acidification. *C. glabrata*-containing phagosomes recruit EEA-1 and LAMP-1 (lysosomal-associated membrane protein-1) marker proteins, indicating normal progression in the early and late endosomal stages, but the biogenesis of phagolysosomes is altered because this pathogen does not acquire cathepsin D (a lysosomal acidic enzyme) and acidification does not occur, allowing fungal replication (Seider et al., 2011). Similarly, in murine macrophages infected with *C. albicans*, the pathogen actively recycles cathepsin D and LAMP-1 out of the phagosomes (Fernández-Arenas et al., 2009). Bain et al. (2015) used live cell imaging to show that *C. albicans* arrests phagosome maturation and acidification. Another mechanism by which *C. glabrata* modulates phagosome maturation was demonstrated using mutant yeasts lacking both the class III phosphoinositide 3-kinase (PI3K) subunit-encoding genes VPS15 and VPS34, which displayed a slightly larger number of acidic phagosomes, suggesting that PIK3 participated in phagosome maturation (Rai et al., 2015).

The deposition of melanin in the cell wall is essential for the pathogenicity of *Cryptococcus* spp. Melanin is formed from L-Dopa and is likely one of the mechanisms responsible for yeast neurotropism (Nosanchuk and Casadevall, 2003). URE1 encodes a urease enzyme, which is involved in the hydrolysis of host and pathogen-produced urea into ammonia, resulting in pH neutralization in the phagosomes of several fungi (Smith and May, 2013). For example, after internalization, *Coccidioides* ssp. is able to resist death via several mechanisms, and urease production and secretion is fundamental for their protection (Mirbod-Donovan et al., 2006). The up-regulation of urease synthesis genes has been noted in the parasitic spherule phase of both *C. posadasii* and *C. immitis* (Whiston et al., 2012).

After phagocytosis of *A. fumigatus*, an unknown mechanism inhibits phagosome maturation, maintaining a neutral pH, and promoting the survival of infective particles until subsequent lysis of the macrophages by the formation of hyphae (Morton et al., 2012). It is known that the DHN-melanin present at the conidial surface is required to avoid lysosomal fusion (Thywißen et al., 2011).

Another mechanism that is shared by different pathogenic fungi, such as *C. neoformans, C. albicans*, and *C. krusei*, is a process called vomocytosis, in which the fungus is cast out of the macrophage without lysis of the host cell (Alvarez and Casadevall, 2006; Garcia-Rodas et al., 2011; Bain et al., 2012). This non-lytic escape is likely to confer advantages to the pathogen by decreasing proinflammatory signals (Gilbert et al., 2015). Little is known about the factors involved in this process. The only evidence reported to date is the participation of the enzyme CnPlb1, the loss of which reduces the process (Chayakulkeeree et al., 2011).

The mechanisms involved in phagosome maturation in *P. brasiliensis* remain unknown. The only study on this subject has been reported by Voltan et al. (2013), who performed an expression analysis of EEA1 and showed an effect on infected macrophages. The authors showed a significant reduction of EEA1 expression after a few hours of infection, resulting in the blockade of trafficking from the trans-Golgi network to phagosomes and the inhibition of phagosome-endosome fusion, suggesting a strategy that is used by *P. brasiliensis* for survival in this environment. Figure 3 summarizes the different fungal mechanisms used to evade the immune system after phagocytosis.

It was recently reported that *C. neoformans* can induce lysosomal damage in infected murine bone marrow-derived macrophages. Consequently, Davis et al. (2015) developed a novel flow cytometric method for measuring lysosomal damage and found that the magnitude of the damage in this organelle is correlated to the increase in *C. neoformans* replication. They also activated the macrophages with IFN-γ to prevent macrophage lysosomal damage and observed an inhibition of *C. neoformans* replication. They concluded that this fungus utilizes lysosome damage as a virulence mechanism to overcome host defense mechanisms and to promote fungal survival; they further suggested that the development of interventions that oppose this ability of *C. neoformans* may be an effective therapeutic strategy.

Numerous reports have suggested that C. neoformans expresses several virulence factors, including the heat shock protein 70 homolog to Ssa1, which occurs through the induction of laccase and can modulate host defense mechanisms. Eastman et al. (2015) determined the effect of Ssa1 in mice infected with a highly virulent serotype A (serA) strain of C. neoformans (H99—Ssa1 deleted) and, surprisingly, noted that, unlike serotype D, H99-serA does not require Ssa1 for laccase expression. The authors further showed that Ssa1 directly promotes early M2 macrophage polarization to improve fungal growth during the innate phase of the immune response.

The interaction between phagocytes and fungi is critical for early control of the infection and thus the ability of the host to clear the infection. Many fungi have developed efficient mechanisms to evade or modulate host cells. Thus, the elucidation of these interactions may contribute to the development of novel immunotherapies to prevent phagocytosis.

## Attack

Pathogens may express on their surface or secrete molecules that directly harm or counter specific host immune defenses. The secretion of toxins or proteases falls into this category (Underhill, 2007). If host defense mechanisms cannot be avoided completely or controlled sufficiently, the last resort for a pathogen is simply to survive or destroy the defenses. To the extent that fungi are robust and hardened against their environments, this is not formally “immune evasion” so much as it is simple survival. However, there are many examples of cases in which fungal pathogens actively destroy or counter specific immune defenses.

### Scavenging oxidative mechanisms

Pathogens are recognized, quickly engulfed, and trapped within an extremely hostile compartment called the phagosome. This organelle is deficient in nutrients and trace elements and undergoes acidification accompanied by increased acidic hydrolase activity. Furthermore, into this organelle are transported a battery of antimicrobial peptides, ROS and reactive nitrogen species (RNS), produced through the NADPH oxidase complex, which combine with nitric oxide (NO) to produce the nitrogen reactive species peroxynitrite. The combined action of these factors has a powerful antimicrobial effect and is normally sufficient to eliminate the pathogen (Nathan and Shiloh, 2000; Babior, 2004).

ROS production by macrophages and neutrophils is a primary mechanism for killing internalized pathogens. A successful pathogenic fungus is one that is able to effectively survive in this powerful antimicrobial environment, resulting in the development of disease. The literature includes many reviews describing some strategies employed by these pathogenic fungi to avoid killing by oxidative stress or antimicrobial mechanisms (Missall et al., 2004; Brown et al., 2014).

The pathogens can choose enzymatic (superoxide dismutases-SODs, catalases-CATs, and peroxiredoxins-PRXs) and non-enzymatic (melanin, mannitol and trehalose) mechanisms to maintain the redox homeostasis within the host cell and resist oxidative stress and/or repair damage. The rapid inductions of mRNAs that encode oxidative stress detoxification and repair proteins have been well-characterized in eukaryotic microorganisms. The transcriptional responses to oxidative stress induce a set of antioxidant enzyme-encoding genes, in addition to genes that encode components of the glutathione/glutaredoxin (GSH, TTR) and thioredoxin (TSA, TRX, TRR) systems, which play critical roles in repairing oxidatively damaged proteins, protein folding, and sulfur metabolism (Missall et al., 2004; Aguirre et al., 2006; Chai et al., 2009).

Several studies have identified individual proteins of the remarkably robust and redundant antioxidant system in different fungi. *C. neoformans* has four CATs, two SODs, glutathione peroxidases, thioredoxin proteins, the inositol phosphosphingolipid-phospholipase C1, and protein kinase C, which are essential for surviving within the oxidative environment of macrophages (Cox et al., 2003; Missall et al., 2005; Missall and Lodge, 2005; Giles et al., 2006; Gerik et al., 2008). The increase in capsule size that occurs during infection by this pathogen provides protection against oxidative stress and antimicrobial peptides (Zaragoza et al., 2008).

Inactivation of detoxifying enzymes, such as SODs, leads to severe attenuation of the virulence, and viability inside immune cells (Fradin et al., 2005; Frohner et al., 2009). A study conducted by Holbrook et al. (2013) evaluated the importance of extracellular and intracellular CAT activity, which presented redundant detoxification activity and facilitated *H. capsulatum* pathogenesis. The same profile was observed for SOD, as shown by Youseff et al. (2012). A study was initiated to characterize *P. brasiliensis* CAT and demonstrated that this protein was induced when the yeast cells were exposed to H_2_O_2_, suggesting that it might be involved in the response to and degradation of this toxic species and thus contribute to the survival of the parasite during infection (Moreira et al., 2004). *Candida* spp. also possess several enzymes that function in a protective manner against the respiratory burst, such CATs, SODs, and glutathione peroxidases (Briones-Martin-Del-Campo et al., 2014).

Campos et al. (2005) and Parente et al. (2015) described the powerful antioxidant defense system possessed by *Paracoccidiodies* spp., which consists of an integration of all the previously described systems. Like so many other fungal pathogens, it uses mechanisms to evade the human immune system, and to survive within infected host cells (Dantas et al., 2008). These features have also been described recently by Tamayo et al. (2016), who showed that the antioxidant enzymes, SODs, assist in combating the superoxide radicals generated during host-pathogen interactions: during the transition process, the fungi are exposed to oxidative agents and interact with phagocytic cells.

NO and its derivatives are important reactive species in the macrophage response to fungal infection. In fact, NO generated by the inducible nitric oxide synthase (iNOS) in mammal hosts exerts a fungistatic effect. Exposure to RNS such as NO causes molecular damage such as S-nitrosylation of the thiol groups of cysteines in proteins and glutathione (Missall et al., 2004; Brown et al., 2014). The enzymes that detoxify RNS have relevant roles in survival and/or virulence in several fungi, including *C. neoformans* (Missall et al., 2006), *H. capsulatum* (Lane et al., 1994), *C. albicans* (Kaloriti et al., 2012), and *Paracoccidioides* spp. (Gonzalez et al., 2000).

Following exposure to NO, *C. albicans* induces increased gene expression. Hromatka et al. (2005) demonstrated that the most highly induced gene is *YHB1*, a flavohemoglobin that combats the RNS stress originating from the developing nitrite (Cánovas et al., 2016) not only in *C. albicans* but also in other fungal pathogens such as *C. neoformans* and *A. fumigatus* (de Jesús-Berríos et al., 2003; Lapp et al., 2014). Furthermore, deletion of this gene results in hypersensitivity to NO and a moderate attenuation of virulence (Hromatka et al., 2005). Other proteins involved in the detoxification of NO are the porphobilinogen deaminase *hemC*, which promotes the activity of flavohemoglobin, the NO-inducible nitrosothionein *ntpA*, which scavenges NO through S-nitrosylation in *A. nidulans* (Zhou et al., 2012, 2013) and S-nitrosoglutathione (GSNO) reductase, which reduces GSNO to ammonia and glutathione disulfide and is important for the detoxification of RNS in *C. neoformans* (Fernández et al., 2003) and *A. fumigatus* (Lapp et al., 2014).

*P. brasiliensis* mutants of cytochrome C peroxidase display increased sensitivity to RNS, and this mitochondrial heme enzyme reduces the peroxy bond of H_2_O_2_ and functions as a heme-based peroxide sensor in yeast mitochondria (Parente et al., 2015).

In addition to combating nitrosative stress, *B. dermatitidis* resists macrophage killing by NO not through detoxification as described above, but rather by suppressing macrophage NO production by interfering with the activity of iNOS (Rocco et al., 2011). In other pathogens such as *P. brasiliensis* and *C. immitis*, NO suppression has also been postulated to occur during infection, with an upregulation of IL-10 that reduces the expression of iNOS and the production of NO, and induction of the host enzyme arginase, which reduces the availability of arginine for iNOS, subsequently reducing the ability of the host to produce nitric oxide, respectively (Hung et al., 2007; Moreira et al., 2010).

In addition to antioxidant enzymes, there are non-enzymatic defenses against ROS and RNS in the form of several metabolites that are important scavengers for detoxification. The ability to produce melanin is one of these defenses. It is known that melanin reacts with most ROS, and acting as a buffer against external ROS, it might function as a sink for potentially harmful unpaired electrons. In fungi, several different types of melanin have been identified, and the two most important ones are DHN-melanin and DOPA-melanin (Jacobson, 2000; Langfelder et al., 2003).

In addition to serving as a reserve carbon source, mannitol is known to scavenge ROS. Mannitol extinguishes reactive oxygen species, prompting speculation that it can assume a cell reinforcement role during host-pathogen interactions. There are reports that during the infection processes, the pathogenic fungus secretes large amounts of mannitol, and a low-producing mannitol mutant exhibited reduced pathogenicity and oxidative stress tolerance (Chaturvedi et al., 1996, 1997; Meena et al., 2015; Erwig and Gow, 2016).

In yeast and filamentous fungi, large amounts of trehalose are stored as a reserve carbohydrate. Trehalose is a non-reducing disaccharide that constitutes up to 15% of the dry weight. It accumulates in response to heat and oxidative stress and has important role as a stress metabolite, stabilizing membranes, and native proteins, as well as by suppressing the aggregation of denatured proteins. It also has distinctive properties such as strong hydrophilicity and chemical stability (Argüelles, 2000; Missall et al., 2004).

Table 2 highlights studies examining pathogenic fungi in relation to mechanisms of protection against oxidative and nitrosative stress, indicating the specific antioxidant agents, and approaches used to identify them.

**Table 2:** **Different studies related to protection against oxidative and nitrosative stress in pathogenic fungi**.

**Pathogenic fungi**	**Antioxidant agent**	**Stress**	**Approach**	**Reference(s)**
*C. neoformans*	Srx1	Oxidative	Deletion constructs and Northern blot	Upadhya et al., 2013
*C. neoformans*	Tsa1	Oxidative and nitrosative	Deletion constructs	Missall et al., 2004
*C. neoformans*	Trx1 and Trx2	Oxidative and nitrosative	Real-time polymerase chain	Missall and Lodge, 2005
*C. neoformans*	Yap1 (a transcriptional factor) that stimulates Trx and Gpx	Oxidative	Mutant strains	Paul et al., 2015
*C. neoformans*	PKC1	Oxidative and nitrosative	Deletion construct	Gerik et al., 2008
*P. brasiliensis*	CAT, SOD, Trx, CCP	Oxidative	Proteomic analysis	de Arruda Grossklaus et al., 2013
*P. brasiliensis*	CAT	Oxidative	Western blot	Moreira et al., 2004
*P. brasiliensis*	CAT, CCP	Oxidative	Enzyme assays, Northern blot	Dantas et al., 2008
*P. brasiliensis*	SOD1, SOD3	Oxidative	Knockdown constructs	Tamayo et al., 2016
*P. brasiliensis*	CCP	Nitrosative	Knockdown construct	Parente et al., 2015
*C. albicans*	CAT, Trx, Tsa, Trr, Gpx, Gsh	Oxidative	DNA microarray	Enjalbert et al., 2006
*C. albicans*	Flavodoxin-like proteins (FLPs)	Oxidative	Mutant construct	Li et al., 2015
*C. glabrata*	Gsh	Oxidative	Mutants constructs	Gutiérrez-Escobedo et al., 2013
*C. albicans*	Cwt1p (acting antagonistically repressing the flavohemoglobin Yhb1p)	Nitrosative	Mutant construct	Sellam et al., 2012
*A. fumigatus*	Skn7 and AfYap1p (transcriptional regulators)	Oxidative	Deletion constructs	Lamarre et al., 2007; Lessing et al., 2007
*A. fumigatus*	SOD1, SOD2	Oxidative	Deletion constructs	Lambou et al., 2010

### Scavenging non-oxidative mechanisms

However, the host may use non-oxidative mechanisms against fungi based on the finding that patients with chronic granulomatosis disease, an inherent disease in which the fundamental genetic defect is in the assembly of NAPDH oxidase, and thus phagocytic oxidative is hampered, have an incidence of aspergillosis ranging from 40 to 70% during their lifetime (Herbrecht et al., 2002; Segal and Romani, 2009). Furthermore, human granulocytes that are deficient in either NADPH oxidase or MPO are incapable of efficient killing of *Candida in vitro* (Lehrer and Cline, 1969; Lehrer, 1970). NADPH oxidase deficiency in patients is associated with significantly increased susceptibility to invasive mold infection, but it has little effect on susceptibility to *Candida* infection. This finding suggests that alternative mechanisms *in vivo* can compensate for a defect in NADPH oxidase-dependent killing mechanisms. Similarly, MPO deficiency in humans does not lead to a predisposition to *Candida* infection unless concomitant risk factors are present (Lanza, 1998; Netea et al., 2015).

Among these mechanisms we can cite the following: extracellular traps ejected by macrophages and neutrophils (METs and NETs), which are web-like structures composed of dsDNA, histones and antimicrobial peptides and proteases (Boe et al., 2015), nutritional stress, cationic stress, and proteases, among others (Brown et al., 2014).

The microenvironment of phagocytic cells is inhospitable before phagocytosis, and during the generation of ROS, the cation levels are also increased. Kaloriti et al. (2012) reasoned that the potency of neutrophils might be due the synergistic combination of oxidative and cationic stress, rather than the additive effects of the individual stresses.

In the macrophage environment, pathogens switch to a gluconeogenic growth mode (shift from fermentative to non-fermentative metabolism during the infective process; Lorenz et al., 2004). The starvation-like response is specific to carbon metabolism and the mutation of genes encoding key steps in gluconeogenesis; the glyoxylate cycle and β-oxidation of fatty acids attenuate virulence to a greater or a lesser degree. The pathogen induces genes in the glyoxylate cycle and uses two-carbon (C2) compounds as a carbon source for gluconeogenesis, such as the products of fatty acid degradation for energy production and survival inside host cells (Barelle et al., 2006). With the increasing population of immunocompromised people, the frequency of invasive fungal infection continues to rise, making the need for effective treatments more imperative. The enzymes of the glyoxylate cycle are valuable targets for the development of antimicrobial drugs because this pathway does not exist in the mammalian host.

ECE1 is a specific hyphal gene encoding a membrane protein that is dependent on the cAMP pathway (Miwa et al., 2004) and was one of the first genes to be identified during hyphal-specific expression. Furthermore, ECE1 is among the most highly expressed genes in the extension of hyphae, displaying increased expression during the course of mycelial growth; however, it does not participate in the initial occurrence of the morphology (Fan et al., 2013). Additionally, this protein may be involved in the formation of biofilms (Bandara et al., 2013). The amino acid sequence of the fungus suggests that it is secreted from hyphae as a group of eight short protein fragments, or peptides, and thus would be well-positioned to interact with host cells. An analysis of synthetic versions of each peptide revealed that one, Ece1-III, elicited the same responses from epithelial cells as hyphae and was denoted “*candidalysin”* (Moyes et al., 2016). Candidalysin directly damages epithelial cell membranes, triggers a danger response signaling pathway and activates epithelial immunity. Membrane permeabilization is enhanced by a positive charge at the carboxy terminus of the peptide, which triggers an inward current concomitant with calcium influx (Moyes et al., 2016).

Upon the initiation of fungal infection, PRRs, especially dendritic cells, recognize fungal pathogens and surfaces and recruit Ly^6^C^*hi*^ monocytes to inflammatory sites during infection. Once in the tissue, these monocytes differentiate into macrophages and inflammatory dendritic cells, including TNF-α and iNOS-producing cells and playing an important role in the control of infection (Serbina et al., 2008; Wüthrich et al., 2012). However, *Blastomyces dermatitidis* have the ability to interfere with Ly^6^C^hi^ recruitment after respiratory vaccination by inducing MMP2 (lung matrix metalloproteinase 2), which suppresses CCL7, one of the signals for monocyte recruitment (Wüthrich et al., 2012). In addition, *B. dermatitidis* DppIVA, which is a multifunctional protein that can act as a serine protease, assists in evasion of the host immune response during infection by cleaving CCL7, a C-C chemokine signal, which recruits Ly6Chi CCR2+ monocytes to the sites of infection (Sterkel et al., 2016). DppIVA also acts on mammalian GM-CSF (granulocyte-macrophage colony-stimulating factor), which is involved in the differentiation and activation of monocytes, macrophages, dendritic cells, and neutrophils during the immune response to pathogens. Inactivation of GM-CSF indirectly affects the production of ROS, increasing the survival of fungi (Sterkel et al., 2016).

## Discussion

Many efforts have been initiated to understand fungal evasion of the immune system. The results of these efforts have shed new light on the diversity and sophistication of the means by which each fungal pathogen subverts the immune system. Some fungi can use more than one strategy to escape immune responses; moreover, sometimes there are different mechanisms within the same species to avoid extermination. While these mechanisms are generally insufficient to overcome a fully intact immune system—hence the rarity of systemic fungal infections—they are likely an important component of pathogenesis in debilitated hosts and represent a fascinating window into the evolution of a complex host-pathogen interaction. The next few years are certain to identify additional means by which fungi modulate immune functions and thus provide new insights regarding challenging questions related to fungal pathogenesis. An understanding of these sophisticated mechanisms of immune evasion can also facilitate the development of novel preventive and treatment therapies to control infection. Conventional antifungal therapy associated with adjuvant immunotherapy appears to be the prominent treatment for fungal infections because failure therapy, rather than the absence of effective antifungal agents, has the highest correlation to ineffective host defense mechanisms. For this purpose, knowledge of the immune system and its interaction with pathogenic fungi is needed, such as the identification of fungal recognition receptors, host defense mechanisms, and cell types involved in these processes, as well as strategies used by fungi to escape the immune system. Understanding the mechanisms by which fungi elude host immune system antimicrobial defense to achieve successful infection could lead to the identification of new drug targets and the development of safe vaccines. Since the availability of antifungal agents is still limited and no vaccine is currently available, this goal is of great importance for the treatment of fungal infections.

## Author contributions

All authors listed, have made substantial, direct and intellectual contribution to the work, and approved it for publication.

### Conflict of interest statement

The authors declare that the research was conducted in the absence of any commercial or financial relationships that could be construed as a potential conflict of interest.
